# Incentive motivation in pet dogs – preference for constant vs varied food rewards

**DOI:** 10.1038/s41598-018-28079-5

**Published:** 2018-06-27

**Authors:** Annika Bremhorst, Sarah Bütler, Hanno Würbel, Stefanie Riemer

**Affiliations:** 1 Division of Animal Welfare, DCR-VPHI, Vetsuisse Faculty, University of Bern, Länggassstrasse 120, 3012 Bern, Switzerland; 2 Animal Behaviour, Cognition and Welfare Group, School of Life Sciences, University of Lincoln, Lincoln, LN6 7DL UK

## Abstract

Recently, there has been a move towards positive reinforcement using food rewards in animal training. By definition, rewards function as reinforcers if they increase or maintain the frequency of behaviour that they follow. However, in operant conditioning tasks animals frequently show systematic changes in performance – in particular a reduction in responding over time. One suggested strategy to avoid such performance decrements is to provide a variety of food rewards, rather than the same food reward in all trials. The enhancement of appetitive behaviour and consumption by reward variation is referred to as ‘variety effect’. We investigated whether dogs preferred a variable or a constant food reward in a concurrent two-choice test. Of 16 dogs, six subjects showed a significant preference for the varied food reward and six for the constant food reward, while four dogs exhibited no significant preference for either option. At the group level, there was a significant effect of block: preference for the varied food reward increased across six blocks of ten trials each. Thus, although some individuals may prefer a single, favourite food reward in the short term, introducing variation in reward types may maintain dogs’ motivation in operant tasks over a longer time period.

## Introduction

Since the Law of Effect first explicitly formulated by Thorndike^1^, stating that responses followed by satisfaction to the animal will be more likely to recur while those followed by discomfort will be less likely to occur again, scientists have been interested in how positive consequences (such as a reward) or negative consequences (such as an aversive stimulus) alter behaviour and operant responding. By definition, rewards function as reinforcers if they increase or maintain the frequency and strength of behaviour that they follow^2^. But not all rewards are equally reinforcing, and additionally shifts in the subjective value of a given reward can be observed.

Traditionally, it was assumed that an animal’s operant responding, i.e. its performance in a task, was directly related to the value of the reward received contingent on the response (see e.g.^3^; reviewed by^4,5^). However, it is now recognised that the relationship between reward value and performance is not straightforward: rather than being an intrinsic property, reward value depends on the individual subject, their previous experiences, current state, and context^6,7^. For example, operant responding may be enhanced when animals unexpectedly receive a larger reward than previously (positive contrast), while a drop in performance may occur following a sudden reward downshift, even below the level shown by animals that have only ever experienced the lower value reward (successive negative contrast effect^5^). Yet, the opposite may also be observed: positive induction leads to an increase in responding upon receipt of a less favoured reward, in anticipation of the preferred reward to follow – similarly as in secondary reinforcement or higher-order conditioning (reviewed by^8^).

Besides possible contrasting effects of different reward values, systematic changes in performance can frequently be observed even when the reward used remains constant. Often, responses increase initially, followed by a gradual decrease as the experimental session progresses^9–11^. Studies have indicated that rather than satiation or fatigue, the processes of sensitisation (defined as “an increase in responsiveness to a repeatedly presented stimulus”^12^) and habituation (defined as “a decrease in responsiveness to a stimulus when that stimulus is presented repeatedly or for a prolonged time”^12^) best explain the observed response patterns to repeated presentation of the same food reward^12^, with sensitisation often, but not always, preceding habituation^9,12^.

In animal training and behaviour modification, the aim is typically to achieve a stable operant performance. One way to counteract late-session decreases in responding lies in providing a variety of food rewards, rather than the same reward in all trials (e.g.^11,13,14^). The enhancement of appetitive and consummatory behaviour by reward variation is referred to as ‘*variety effect’*^8,13^. A number of studies have demonstrated that variety effects manifest not only in an increased consumption when variable food types are available, but also in operant responding. Thus, rats show higher response rates in lever pressing tasks when reinforcement is variable (one of three different rewards is delivered unpredictably) than when it is constant (every response is followed by the same reward)^15^. Likewise, responding is highest when two different food types are presented intermixed compared to either food type presented alone^16,17^. On this basis, Steinman^16^ concluded that varied reinforcement can increase response rates, as well as leading to greater resistance to extinction of an operant behaviour. This finding has since been replicated in further studies using operant paradigms in rats^13,14^ and dwarf hamsters^11^, as well as in human adults^18^, normally developing children^9^, and autistic children^19^. Several properties of habituation can explain the ‘*variety effect’* phenomenon, including (I) stimulus specificity, i.e. disruption of habituation when a novel food is provided, (II) slower habituation due to an increased interval between successive presentations of the same food, (III) repeated dishabituation as the stimulus is repeatedly changed, and (IV) spontaneous recovery when the habituated stimulus is absent^12,13^.

While only a single reward option at a time was available in the above-cited studies (i.e. only the same food reward or only varied food rewards), a few studies have explored subjects’ preferences when given the choice between constant or varied food rewards concurrently. In Milo and colleagues^20^, four autistic boys demonstrated a preference for varied over constant rewards in both a single choice test (baseline condition) and a concurrent choice test where pressing of one button led to access to a constant reward and pressing of another button led to delivery of one of three reward options, presented in a random order^20^. Preference for the varied versus the constant reward option was less clear in a study on seven children diagnosed with mental retardation: four participants responded more or longer for the varied reward, two preferred the constant reward and one individual responded equally to both options^21^. Conceivably, the preference for the varied reward was less consistent in this study because the subjects’ most favoured reward was available only in the constant condition^21^.

Similarly, a preference for the food provided in the constant condition could potentially explain performance in a study in which six gilts were tested in a two-choice task where one lever was associated with a constant food reward and the other was associated with unpredictable varied reward (one of eight different food items, including the food used in the constant reward option)^22^. Contrary to the predictions defined in this study, four of six pigs preferred the predictable constant option, one the unpredictable varied option and one exhibited no significant preference^22^. To our knowledge, no other study to date has attempted to “ask” nonhuman animals about their preferences for constant vs varied food reward when given the choice.

Giving animals the possibility to choose their reward themselves has the advantage of allowing them to select the reward in line with their current motivational state, which may change over time^21^. Asking animals for their food preference choices provides important insights for the use of food rewards when training animals in experimental studies and beyond. Besides learning theoretical considerations, the question of whether animals would prefer varied or constant food rewards is also of practical relevance, given that recent decades have seen a move towards positive reinforcement using food rewards in animal training. Applications range from voluntary participation in medical procedures and husbandry training in laboratory^23^ and zoo animals^24–28^, cognitive enrichment in farm animals^29^, and from effective and humane training of pet dogs (e.g.^30^) to advanced training of working dogs (for example guide dogs for the blind^31^, assistance dogs for humans with hearing or mobility impairments^32^, therapy dogs^33^, military dogs^34^, police dogs^35^, search and rescue dogs^36^, and water rescue dogs^37,38^).

A few studies have established that most dogs appear to prefer food rewards to praise or petting^39–41^ and, comparing these three reward types, food has been shown to reduce the number of sessions required to learn the response to a verbal command in early but not in later stages of training^40^. Nonetheless, how to use this reward type most effectively in training has rarely been investigated (but see^42–44^). With the present study we aimed to test whether domestic dogs show evidence of a ‘variety effect’ when given the choice between a constant and a varied food reward option. To establish dogs’ food preferences, dogs were first tested with three food types in a food preference test. In a subsequent two-choice task, dogs could select either a constant reward option (the preferred food type according to the preference test) or a varied reward option (semi-random presentation of all three food types). After learning the contingencies of the two response options, dogs were given 60 trials (separated into 6 blocks within a single session) in which they could choose between the two options.

On the basis of previous findings indicating habituation effects to constant food rewards and enhanced performance when they are varied (e.g.^9,11,13,14,16,18,19^), we hypothesized that this effect manifests in the subjects’ preferences when they are able to select between the two options. Therefore, we predicted firstly that dogs prefer the varied reward option and secondly that preference for the varied reward becomes more pronounced with progressing blocks, reflecting habituation.

## Results

### Food Preference Test

Three food types (sausage, cheese and a commercial liver dog treat) were used. Following tasting of each food type, one piece of each food was placed under one of three equidistant wire grids so that dogs could see and smell, but not access the food. Time spent directing behaviour at the three grids was used as a proxy for determining relative preference for the three food types for each subject^45^.

All dogs consumed all the food types in the first part of the preference test. During the inaccessible condition, they spent on average 42.58 s (70.9%) of the time directing behaviours at the grid covers of the three food types. 43.75% of dogs preferred sausage, 31.25% preferred the cheese and 25% preferred the dog treat, with proportion of time spent interacting with the preferred food type ranging from 33.84% to 76.49% (Supplementary Table S1).

### Concurrent two-choice test for a variable vs a constant food reward option

At the group level, dogs’ choices for either of the two options were not significantly different from chance, both when evaluating all 6 blocks together (t_14_ = −0.071, p = 0.944) and when separated into the first and the second half of trials (t_15_ = −0.874, p = 0.396 and t_14_ = 0.331, p = 0.745, respectively).

**Figure 1:**
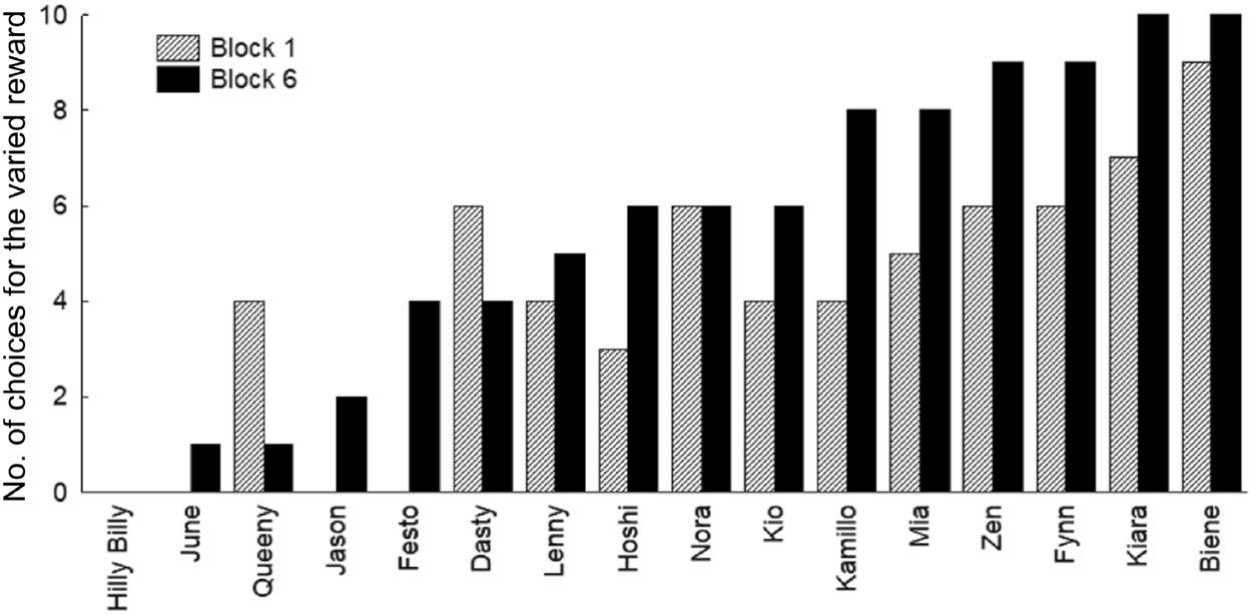
Number of choices for the variable food reward option in the first vs the last block (dogs ordered according to strength of preference for the varied food reward option). One dog (June) completed only four blocks and is therefore not included in the figure.

The linear mixed models, however, demonstrated a significant effect of block (F_1,73_ = 6.278, p = 0.014), with dogs showing a greater preference for the varied reward condition during later blocks. To illustrate this point, Fig. 1 shows a comparison of each individual dog’s choices for the varied food reward option during the first and the last block. See Supplementary Table S2 for raw data for each block.

Neither the effects of side (F_1,73_ = 0.544, p = 0.463), target colour (F_1,73_ = 3.159, p = 0.097), initial strength of preference for the favoured food (F_1,73_ = 0.054, p = 0.813), nor the interaction between strength of preference and block (F_1,73_ = 2.515, p = 0.117) were significant. There was also no significant sex difference (F_1,73_ = 0.097, p = 0.755).

**Figure 2:**
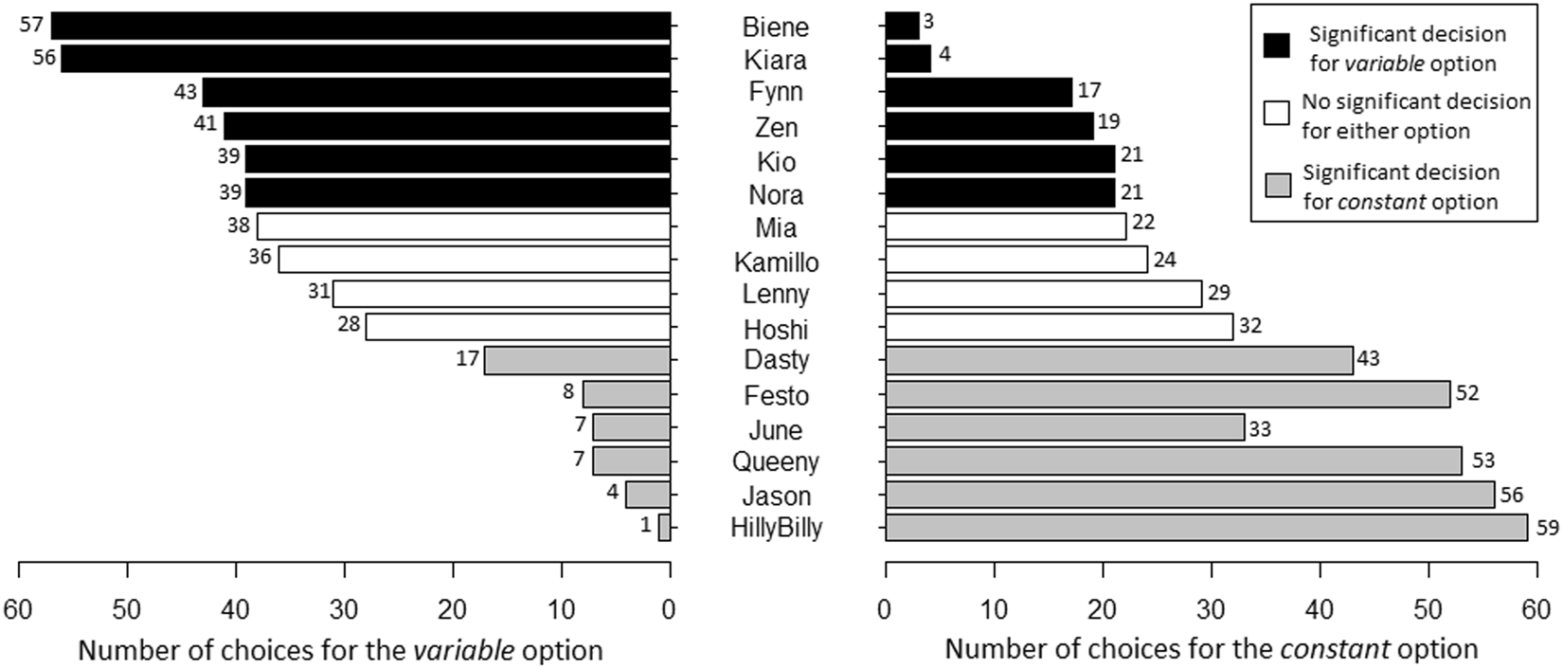
Number of choices for the variable and the constant option, respectively, in the sixteen subjects.

At the individual level, twelve of the sixteen subjects exhibited a significant preference for one of both food reward options in the choice test, with six dogs preferring the constant reward and six dogs preferring the varied reward option (Fig. 2, Table 1).

**Table 1:** Results from the two-choice test for all 60 test trials, including the more frequently chosen option (in parentheses if non-significant), the covariates, number of choices for the variable option, proportion of choices for the variable option, and the p-value of the binomial test.

Dog ID	More frequently chosen option	Colour of constant option	Side of constant option	Frequency of choosing variable option (of 60 trials)	% of choosing variable option	Binomial test p-value
HillyBilly	constant	yellow	right	1	1.67	<0.0001*
Jason	constant	yellow	left	4	6.67	<0.0001*
June^♦^	constant	yellow	left	7	17.5	<0.0001*
Queeny	constant	yellow	left	7	11.67	<0.0001*
Festo	constant	yellow	left	8	13.33	<0.0001*
Dasty	constant	blue	right	17	28.33	0.0011*
Hoshi	(constant)	yellow	left	28	46.67	0.6989
Lenny	(variable)	yellow	right	31	51.67	0.8974
Kamillo	(variable)	blue	left	36	60	0.1550
Mia	(variable)	yellow	left	38	63.33	0.0519
Kio	variable	blue	left	39	65	0.0273*
Nora	variable	blue	right	39	65	0.0273*
Zen	variable	blue	right	41	68.33	0.0062*
Fynn	variable	blue	left	43	71.67	0.0011*
Kiara	variable	blue	right	56	93.33	<0.0001*
Biene	variable	yellow	right	57	95	<0.0001*

An asterisk indicates a significant effect after FDR correction (with p < 0.05). Subjects ordered by frequency of choosing the variable option. ^♦^This dog completed only 40 test trials.

Given the significant effect of block at the group level, individual-level analyses were additionally performed separately for the first and the last 30 trials. In the first 30 trials only two of 16 dogs exhibited a significant preference for the variable option, one had a tendency to prefer the variable option (p < 0.1), and six dogs significantly preferred the constant option, with the remaining dogs showing no preference (Table 2). Meanwhile, in the last 30 trials, the number of dogs significantly preferring the variable option increased to five, with two dogs showing a tendency towards the variable option, five dogs preferring the constant option and three dogs demonstrating no clear preference (one dog only completed only 40 trials and was therefore not included in the analysis of the last 30 trials) (Table 2).

**Table 2:** Results from the two-choice test, separated into the first and the last 30 trials, including the more frequently chosen option (in parentheses if non-significant), number of choices for the variable option, and the p-value of the binomial test.

Dog ID	First 30 trials			Last 30 trials		
	More frequently chosen option	Frequency of choosing variable option	Binomial test p-value	More frequently chosen option	Frequency of choosing variable option	Binomial test p-value
Hilly Billy	constant	1	<0.0001*	constant	0	<0.0001*
Jason	constant	2	<0.0001*	constant	2	<0.0001*
June^♦^	constant	3	<0.0001*	NA	NA	NA
Queeny	constant	5	0.0003*	constant	2	<0.0001*
Festo	constant	2	<0.0001*	constant	6	0.0014*
Dasty	constant	9	0.0427	constant	8	0.0161*
Hoshi	(constant)	11	0.2005	(variable)	17	0.5847
Lenny	(variable)	18	0.3616	(constant)	13	0.5847
Kamillo	(variable)	16	0.8555	(variable)	20	0.0987
Mia	(variable)	16	0.8555	variable	22	0.0161*
Kio	(variable)	19	0.2005	(variable)	20	0.0987
Nora	(variable)	20	0.0987	(variable)	19	0.2005
Zen	(variable)	17	0.5847	variable	24	0.0014*
Fynn	(variable)	18	0.3616	variable	25	0.0003*
Kiara	variable	26	<0.0001*	variable	30	<0.0001*
Biene	variable	27	<0.0001*	variable	30	<0.0001*

An asterisk indicates a significant effect after FDR correction (with p < 0.05). Order of subjects as in Table 1. ^♦^This dog completed only 40 test trials.

## Discussion

On the basis of previous studies on the variety effect in operant responding in humans and nonhuman animals, we predicted that dogs would demonstrate a preference for the varied reinforcement option. The prediction was confirmed for six of sixteen dogs, while six other dogs chose the constant option significantly more often and four dogs exhibited no significant preference at all. Thus, at the group level, dogs did not show the predicted preference for the variable reward option – although it could be argued that a lack of a significant choice of either side observed in four dogs also indicates a preference for the variable condition, since when dogs switched between the two options, exposure to the constant reward type was regularly interrupted in those trials where they selected the varied option. With one exception (one dog selecting the variable option only once in 60 trials), also those dogs with a significant preference for the constant reward option made several choices for the varied option, particularly in later blocks. This intermittent reinforcement with a different food type may be sufficient to maintain variety and cause dishabituation^10^. Thus, these dogs have received varied reinforcement even if the frequency of choices of the varied option was not significantly different from chance.

Our second prediction was an increased preference for the varied reward as the session progressed, which was supported by our results. Indeed, consistent with the effect of habituation to a specific food type^12^, the great majority of dogs (N = 12) selected the varied option more frequently in the last block than in the first block (of the remaining four subjects one selected the varied option equally frequently in both blocks, one never selected the varied option, and two chose the variable option more frequently in the first block; as shown in Supplementary Table S2 and Figure 1).

When taking into account only the individual analysis, given the equal distribution of significant choices for the constant and the varied option, it cannot be ruled out that dogs’ choices simply reflected a side preference. Side preferences commonly develop in two-choice tasks when animals are unable to understand the contingencies (e.g.^46–49^). However, the fact that nearly all dogs were more likely to select the varied option in later trials – including those who significantly preferred the constant option – argues against this possibility. If anything, side biases should become more consistent and not less consistent over time (e.g.^46,50,51^), and trends in side preferences (such as an increase or decrease over time) should be the same for both groups (dogs preferring constant and variable rewards, respectively). Conversely, choices of the preferred option decreased across trials in those dogs exhibiting a preference for the constant food reward but increased in those dogs preferring the varied food reward.

While the increased preference for the varied food reward in later trials is indicative of habituation to the constant reward, this did not translate into a preference for the varied food reward as clearly as in some previous studies – even though our sessions were relatively long (lasting approximately 45–60 minutes, with 20 forced choice trials and 60 test trials in a single session), compared to a session length of only between 20–30 minutes in other studies (e.g.^9,11,13,16,17^) or until a maximum of 50 rewards were delivered^20^ (but see^14^ with 60 minutes sessions and a mean number of up to 197.4 rewards obtained per session). It cannot be ruled out that the relative volume of food ingested by the dogs was lower compared to that consumed by subjects in previous studies, but to our knowledge such measures (e.g. volume or calorific content relative to body weight) have not been reported.

One factor that might have contributed to the relatively high frequency of choosing the constant reward in many dogs is the fact that the foods on offer were of very high value, i.e. the stimulus intensity was high. Habituation normally occurs faster and is more pronounced with weaker stimuli^12,14,52^; therefore the use of highly preferred foods may have slowed habituation, and it is conceivable that a more pronounced preference for the varied reward would be observed with rewards of lower value. In animal studies on the effect of reward variety on operant responding, the relative quality of rewards was often not assessed (but see^13^). If these animal studies used less favoured food types, this could possibly explain the reported stronger manifestation of a variety effect^11,14,16,17^. Studies performing concurrent choice tests like the present study in human children similarly used rewards that were preferred by the subjects in preliminary preference assessments, but with mixed results: (^21^ – preference for varied reward; ^20^ – individual preferences for either varied or constant reward).

Thus, conclusions may differ somewhat between concurrent choice tests – with choice as the dependent variable – and single operant tests – with response rate as dependent variable. While only few studies have investigated animals’ or humans’ choice behaviour for constant vs varied reinforcement, the existence of a variety effects appears to be less unequivocal in tests where the individuals’ choices were assessed^20–22^ than when observing response rates in single-operant arrangements^11,13,14,16^. One explanation for this is that individuals may initially prefer their favoured reward, hence selecting the constant option, and only gradually shift to the varied option as habituation to the preferred reward type sets in, as was observed in the current study.

This study shows pronounced individual differences in preferences for both food reward type (sausage, cheese, and dog treat) and food reward option (constant vs variable). While an increase in preference for the varied reward was observed over time, overall individuals’ preferences for either the varied or constant food reward were expressed quite consistently across blocks. Individual variation in reward-related responses has been observed in a variety of species such as in pigeons^53^ or in rats^8^. In paradigms similar to the current one, marked individual differences were observed, e.g. in pigs^22^ and in human children^21^. Interestingly, while the current study (to our knowledge) is the first study to investigate preference for variety in food reward types in dogs, a previous study on dogs’ preferences for variation in delay to reinforcement yielded similar figures as the current study, with approximately one third of dogs preferring the predictable constant option, another third preferring the unpredictable varied option, and the last third being indifferent^54^.

Most dogs did not seem to base their choice on the energy content of the food, as it was the least energy-dense food (sausage) that was preferred by the majority of dogs (N = 7). A wide variety of factors has been identified as influencing individual food preferences in dogs and other mammals, reflecting a complex interaction between biological tendencies and environmental influences^55^. These include genetic factors, prenatal and postnatal experiences, and while the early ontogeny is particularly influential for the development of food preferences, preferences continue to change during adolescence and even adulthood^55^. In the case of dogs, perinatal (prenatal and early postnatal exposure through the mother’s milk) flavour exposure^56^, housing (pet dogs vs laboratory dogs) and other keeping conditions^57^, size^57^, sex^57^, and social learning^58,59^ are suggested to affect food preferences. Additionally, dogs often display a ‘novelty effect’, i.e. they prefer a different diet to the one they are used to, although neophobia (initial rejection of unfamiliar foods) may also occur^60^.

In humans, it has been demonstrated that individual differences in food habituation are associated with (risk of) overweight in both children^9,61^ and adults^62^, and that the propensity for habituation is a temporally stable trait over one year^63^. In dogs, a deletion in the canine proopiomelanocortin gene has been found to be associated with body weight and greater food motivation in Labrador retrievers and Flat coated retrievers, and additionally with adiposity in the former^64^. In the case of dogs, food intake is largely controlled by the owners, and all of the dogs in the sample had a healthy weight. It is, however, possible, that under free-feeding conditions we would have observed a relationship between a higher body weight and choice of the constant reward (not tested here), since slow food-specific habituation is a risk factor for developing obesity. The relevance of such individual differences for trainability, but also health problems, and associated genetic and environmental variables should be addressed in future studies.

Furthermore research is warranted to compare relative effort when only a single choice is available (either constant or varied food rewards), or when rewards of lower quality are used. Additionally, the effect of intermittent presentation of stimuli other than different food types could be investigated, since stimuli and events unrelated to food can also serve as dishabituators and thus promote a higher response rate for the same food reward^65^. In the case of domestic dogs, one possibility might be to intersperse training with a play session (see also^66^).

The findings of the present study are of importance for training of both pet and working dogs. Dogs are sensitive to reward quality and will adjust their operant behaviour accordingly (e.g.^42,44^). Nonetheless, the current study suggests beneficial effects of variation in reward types on motivation over longer time periods. Desirable consequences of optimising the efficiency of food rewards in everyday use may include enhanced motivation of dogs to cooperate, better obedience, fewer undesirable behaviours, and improved working dog performance.

## Conclusions

Although dogs exhibited no significant preference for either constant or varied food rewards at the group level, the occasional choices of the varied option even in dogs with an overall preference for the constant reinforcement likely promoted dishabituation to the constant food reward, and thus maintained the effectiveness of this reward type. Additionally there was a significant effect of block, with preference for the varied reinforcement increasing as the blocks progressed. Thus, although some individuals may prefer a single, favourite food reward in the short term, introducing variation in food reward types may maintain dogs’ motivation in operant tasks over a longer time period.

## Methods

The study was assessed and approved by the cantonal authority for animal experimentation, the Veterinary Office of the Canton of Bern (Switzerland) (Licence number BE29/17) and complies with the “Guidelines for the Treatment of Animals in Behavioral Research and Teaching” of the Association for the Study of Animal Behavior (ASAB).

### Subjects

Subjects were eighteen privately owned pet dogs of both sexes, various breeds and ages (range 2–12 years; Supplementary Table S3). All owners gave written consent to their dogs’ participation. One dog failed to complete pre-training due to lack of motivation, and another dog was excluded from the analysis due to experimenter error when designating the favoured food type. Thus, the final sample comprised sixteen dogs of which half were female (of which five were neutered) and half were male (of which seven were neutered). Most of the subjects were very well trained (e.g. obedience, agility, trick dog, dummy training). Two of the dogs were trained as guide dogs for the blind. 13 of the 16 dogs had previous experience with clicker or marker training.

### Food types

Three food types, considered to be of very high value for most dogs, were used: cheese (M-Budget Gouda, 28% fat, 23% protein), sausage (Micarna Tierfutterwurst gekocht 7.1% fat, 12.3% protein) and a commercial dog treat containing liver (Asco Softy Bits mit Leberwurst, 12% fat, 24% protein). All owners confirmed that their dogs did not have intolerances against or allergies to any of these food types.

#### Food preference test

The dogs were tested in a room measuring 5.5 m × 7 m at the campus of the Vetsuisse Faculty, University of Bern (Switzerland). To establish a relative preference for the three food types for each dog, all dogs participated in a food preference test during the first appointment. The test was adapted for three food types from^45^, who demonstrated that time spent trying to access two different inaccessible food rewards corresponded to consumption measures when these two food types were concurrently available.

Initially, the dog was held between the owner’s legs for this test. For smaller dogs, the owner was sitting on a chair, for larger dogs the owner was standing. First, the experimenter gave the dog one piece of each food type (all of approximately the same volume) to taste in a random order. A piece of each food type was then placed in one of three identical bowls, located in a semi-circle 0.75 m from each other and all equidistant 1.5 m from the dog’s starting point, with the location of the food types counterbalanced between subjects. A wire grid nailed onto a heavy wooden board (so that it could not be moved by the dogs) was located behind each bowl (to be used in the second, ‘blocked’, part of the test). The dog was guided on lead to each bowl to consume the food piece, with half the dogs starting with the left bowl and half the dogs starting with the right bowl and continuing either counter-clockwise or clockwise (counterbalanced). In the third step, the bowls were refilled with a piece of the same food type as before. Every bowl was then placed under the grid cover such that it was inaccessible to the dog. The dog was released and given one minute to explore the three inaccessible food bowls. The owner and the experimenter ignored the dog during this time.

Tests were videotaped and behaviour directed at the three grid covers preventing access to the different food types were coded from the videos, following the methodology in^45^. Time investigating a food type was counted if the dog showed any of the following behaviours: looking, sniffing, pawing at and vocalizing towards a cage^45^. On the basis of total investigation time of each food type, we generated an order of preference for the three food types for each dog. To obtain a measure of preference strength, we also calculated the proportion of total investigation time spent directed at the ‘preferred’ reward (Supplementary Table S1).

#### Concurrent two-choice test for a variable vs a constant reward option

**Figure 3:**
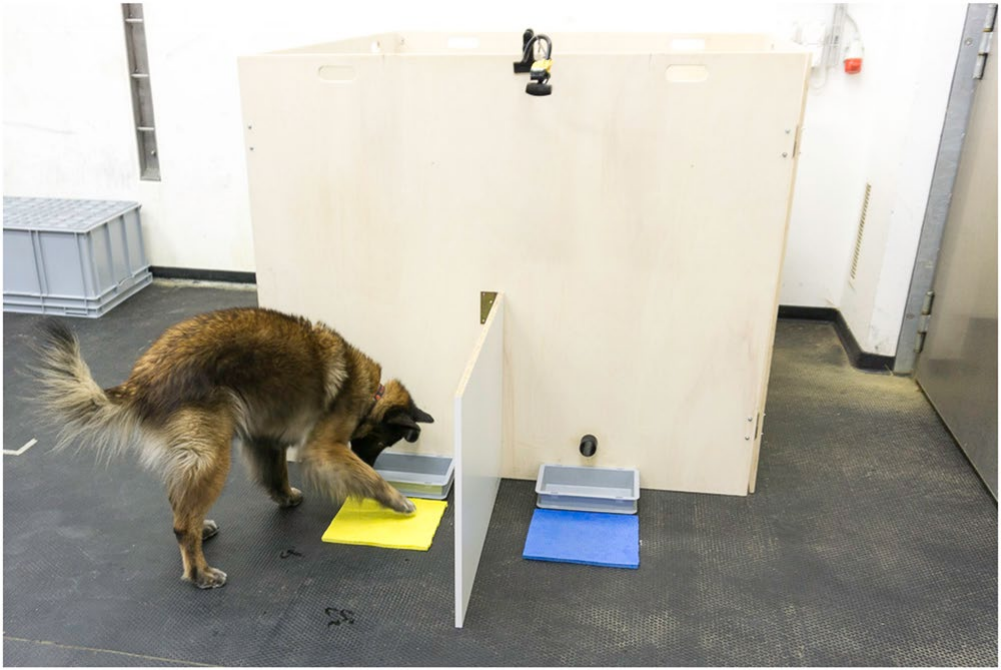
Test setup showing the test apparatus with the two coloured targets, boxes to catch the treats, food delivery tubes, central divider, and overhead camera. The experimenter is not visible behind the wooden wall.

**Test apparatus**: The experimenter was situated in a square wooden enclosure consisting of four solid wooden boards (1.50 m × 1.25 m). On the front wall, two plastic tubes, located 10 cm above the floor and 50 cm apart (25 cm left and right from the centre) projected to the outside and were used to deliver the respective rewards. A flat grey plastic box was fixed under each tube to catch the delivered treat. In front of these boxes, a yellow and a blue square plastic target (25 cm × 25 cm) were placed, with sides of the two colours counterbalanced between dogs. To prevent dogs from switching after making a choice, a wooden barrier of 0.60 m height and 0.80 m length was installed at the centre of the front wall at a right angle to the wall (Fig. 3). Inside the apparatus, the experimenter was not visible to the dog and observed the dog’s behaviour via a webcam (Type Multicam WF-10, HD), installed centrally on top of the test apparatus.

**Choice options:** For the constant option, the food reward that was preferred by the dog according to the preliminary preference test was delivered in all trials. For the varied option, one of the three rewards (including the favoured reward) was delivered in a semi-random order, with the condition that no reward type was delivered more than twice in a row.

**Pre-training:** Pre-training was performed to familiarise the dogs with the test procedure, but away from the test apparatus to be used in the actual test. Using a mix of shaping and/or luring, with semi-dry dog food as a reward, dogs were first trained to touch a square plastic target (25 cm × 25 cm) on the floor with the paw. This training target was identical to the targets used in the concurrent two-choice test, but of a different colour (green). In a second step, dogs were trained to take up a starting position between the handler’s legs and to run forward to the floor target upon a verbal release. When the dogs performed these behaviours fluently (depending on the dog’s previous experience with targeting, this was achieved within one to two sessions, with one fearful dog taking three sessions), they proceeded to familiarization trials at the test apparatus.

**Procedure:** Trials started with the dog between the handler’s legs (all but three subjects were handled by their owners, with three dogs handled by a second experimenter). As above, handlers of smaller dogs were in a sitting position while handlers of larger dogs were in a standing position. By starting centrally between the owner’s legs, we could ensure that the start location was central and so no side bias was induced by the dog’s starting position. The starting point was adjusted based on the size of the dog such that the dog’s nose was 50 cm from the wooden divider between the two choice options.

For half the dogs, the right side was associated with the varied reward option and for the other half the left side was associated with the varied reward option. Likewise, the colours of the targets and associated reward type were counterbalanced (Table 1). For each individual dog, the contingencies remained constant throughout the experiment.

When the dog had looked at the setup for three seconds, the handler released the dog. As soon as the dog touched a target with at least one paw, the experimenter delivered the reward associated with this target through the respective tube. After each trial, the handler led the dog back to the starting position.

**Familiarization trials – forced choice trials:** Dogs received between 60 and 140 forced choice trials (mean = 71.43; SD = 21.79) in order to learn the procedure and the contingencies. During forced choice trials, only one target was available in front of the test apparatus, and so dogs could only earn the reward type associated with that target. Trials were performed in blocks of ten, with half the dogs receiving the constant condition first and the other half receiving the varied condition first. After each block of forced choice trials, the presented target associated with side and reward option was changed, so that the dogs received alternating blocks of the constant and the varied option.

To proceed to testing, dogs had to firstly complete at least 60 familiarization trials (this was considered as sufficient for the dogs to learn the contingencies based on a pilot study where the two sides were associated with a preferred and a non-preferred food reward, respectively, and so a choice for the preferred reward could be predicted) and secondly, they had to approach the target immediately upon release and directly in all trials of the last two consecutive blocks.

Eleven of the sixteen dogs reached this criterion within 60 trials (six blocks). For dogs that had a break of more than two weeks between training sessions (N = 2), that required more trials to learn the procedure (N = 2) or that initially reacted fearfully to the movement of the treat (N = 1), a larger number of familiarization trials (max. 140, 14 blocks) was performed until reaching criterion.

**Test trials – free choice trials:** Test trials were always performed on a separate day. Prior to testing, dogs were given ten forced choice trials on each side to remind them of the contingencies, starting at the same side as the familiarization trials. The 20 forced choice trials were then followed by six blocks of 10 free choice trials in which both targets were available. Except for one dog, which completed only four test blocks due to experimenter error, all dogs received six test blocks on the same day, thus performing a total of 80 trials including the familiarization trials in this session. The dogs’ choices were immediately noted down by the experimenter and additionally videotaped with a Panasonic camcorder HC-V777. To assess reliability of the live recording, sixteen randomly selected blocks of ten trials were later coded from the videos. Correspondence with live recording was excellent, with agreement for 158/160 trials (98.75%).

### Coding and statistical analysis

The preference test videos were coded using Solomon Coder beta 17.03.22 (© András Péter). Statistical analyses were performed in Statistica 6.1 (Statsoft Inc. 2004) and R version 3.3.3 (R Development Core Team 2017).

As the data met the requirements of parametric testing, one-sample t-tests were performed to test whether the dogs’ choices were significantly different from chance at the group level. Subsequently linear mixed effect models (R package nlme, function lme) were calculated to evaluate other factors potentially influencing the dependent variable and proportion of choices for the variable option. Strength of preference for the preferred food (as determined by percentage of time spent investigating this food type in the initial preference test), side and colour associated with the variable condition, block, and the dog’s sex were included as fixed factors, and dog ID as a random factor. Residuals of the model were assessed for meeting the assumptions of parametric testing and were adequate.

To assess whether individual dogs showed a significant preference for one of the two choice options, binomial tests were performed, and False Discovery Rate (FDR) control^61^ was used to correct for multiple comparisons.

## Electronic supplementary material

Supplementary tables 1-3
