# Supplementary material for: Incentive motivation in pet dogs – preference for constant vs varied food rewards
Source: Sci Rep. 2018 Jun 27;8:9756. doi: 10.1038/s41598-018-28079-5 (PMC6021384; doi:10.1038/s41598-018-28079-5)
Supplement: Supplementary file 1 — Supplementary tables 1-3 [file 41598_2018_28079_MOESM1_ESM.docx]

**Incentive motivation in pet dogs – preference for constant vs varied food rewards**

**Bremhorst, Annika^1,2^, Bütler, Sarah^1^, Würbel, Hanno^1^, Riemer, Stefanie^1,^***

^1^ Division of Animal Welfare, DCR-VPHI, Vetsuisse Faculty, University of Bern, Länggassstrasse 120, 3012 Bern, Switzerland

^2^ Animal Behaviour, Cognition and Welfare Group, School of Life Sciences, University of Lincoln, Lincoln LN6 7DL, UK

* riemer.stefanie@gmail.com

**Electronic supplementary material**

**Supplementary Table S1.** Results of the preference test, including preferred food, time spent directing behaviour at the different food types, total interaction time for all three covers and the proportion of interaction time spent directing behaviour at the preferred food.

|  |  | | **Time (s) directed at** | | | **Total time interacting with grid covers (s)** | **% interaction time directed at preferred food** |
| --- | --- | --- | --- | --- | --- | --- | --- |
| **Dog ID** | **Preferred**  **food** | **cheese** | | **dog treat** | **sausage** |  |  |
| Biene | sausage | 15.8 | | 12.6 | 19 | 47.4 | 40.1 |
| Dasty | sausage | 8 | | 6.6 | 8.2 | 22.8 | 35.1 |
| Festo | sausage | 6.6 | | 10.6 | 24.8 | 42 | 59 |
| Fynn | dog treat | 0 | | 15.6 | 9.6 | 25.2 | 61.9 |
| HillyBilly | cheese | 26.2 | | 14.4 | 10.8 | 51.4 | 51 |
| Hoshi | sausage | 10.4 | | 12.8 | 15 | 38.2 | 39.3 |
| Jason | cheese | 13.6 | | 12.6 | 6 | 32.2 | 42.2 |
| June | cheese | 18.6 | | 12 | 15.8 | 46.4 | 40.1 |
| Kamillo | sausage | 8.6 | | 5 | 22.8 | 36.4 | 62.6 |
| Kiara | dog treat | 12.2 | | 15.4 | 12.4 | 40 | 38.5 |
| Kio | cheese | 28.8 | | 8 | 14.8 | 51.6 | 55.8 |
| Lenny | dog treat | 0 | | 43.6 | 13.4 | 57 | 76.5 |
| Mia | dog treat | 11.4 | | 14.8 | 13.4 | 39.6 | 33.8 |
| Nora | cheese | 42.6 | | 12.6 | 1.8 | 57 | 74.7 |
| Queeny | sausage | 11.2 | | 14.8 | 22.6 | 48.6 | 46.5 |
| Zen | sausage | 13.4 | | 6.6 | 25.4 | 45.4 | 55.9 |

**Supplementary Table S2.** Number of choices made by each individual for the variable option in blocks 1-6.

| **Dog ID** | **Block 1** | **Block 2** | **Block 3** | **Block 4** | **Block 5** | **Block 6** |
| --- | --- | --- | --- | --- | --- | --- |
| Biene | 7 | 10 | 10 | 10 | 10 | 10 |
| Dasty | 4 | 2 | 3 | 2 | 1 | 5 |
| Festo | 0 | 1 | 1 | 1 | 1 | 4 |
| Fynn | 6 | 4 | 8 | 7 | 9 | 9 |
| Hilly Billy | 0 | 1 | 0 | 0 | 0 | 0 |
| Hoshi | 3 | 4 | 4 | 5 | 6 | 6 |
| Jason | 0 | 0 | 2 | 0 | 0 | 2 |
| June^♦^ | 1 | 0 | 2 | 4 |  |  |
| Kamillo | 4 | 5 | 7 | 6 | 6 | 8 |
| Kiara | 9 | 7 | 10 | 10 | 10 | 10 |
| Kio | 6 | 6 | 7 | 7 | 7 | 6 |
| Lenny | 6 | 7 | 5 | 5 | 4 | 4 |
| Mia | 5 | 4 | 7 | 6 | 8 | 8 |
| Nora | 4 | 10 | 6 | 7 | 6 | 6 |
| Kio | 4 | 1 | 0 | 1 | 0 | 1 |
| Zen | 6 | 5 | 6 | 7 | 8 | 9 |

^♦^ This dog completed only 40 test trials

**Supplementary Table S3.** Name, breed, sex, neuter status and age (years) of the subjects.

| **Dog ID** | **Breed** | **Sex** | **Neuter status** | **Age** |
| --- | --- | --- | --- | --- |
| Biene | Spanish Water Dog | female | neutered | 9 |
| Dasty | Rhodesian Ridgeback | female | neutered | 7 |
| Festo | German Shepherd | male | neutered | 2 |
| Fynn | Border Collie | male | neutered | 7 |
| HillyBilly | Tervuren | female | neutered | 9 |
| Hoshi | Shiba Inu | male | neutered | 6 |
| Jason | Australian Shepherd | male | neutered | 9 |
| June | Australian Shepherd | female | intact | 4 |
| Kamillo | Spanish Water Dog | male | intact | 3 |
| Kiara | Standard Poodle | female | intact | 12 |
| Kio | Shiba Inu | male | neutered | 12 |
| Lenny | Podenco cross | male | neutered | 9 |
| Mia | Mixed breed | female | neutered | 2 |
| Nora | German Shepherd cross | female | neutered | 11 |
| Queeny | Standard Poodle | female | intact | 8 |
| Zen | Labrador Retriever | male | neutered | 2 |
